# Characterization of Key Aroma Compounds in Botanical Lujiu Based on Jiang‐Flavor Baijiu by Comparative Aroma Extract Dilution Analysis, Quantitative Measurements, and Aroma Traceability Analysis

**DOI:** 10.1002/fsn3.71222

**Published:** 2025-12-26

**Authors:** Yongsu Li, Yubo Yang, Bo han Zhang, Fan Yang, Ping Xiang

**Affiliations:** ^1^ Kweichow Moutai Co., Ltd Renhuai Guizhou China; ^2^ Kweichow Moutai Group Renhuai Guizhou China

**Keywords:** aroma extract dilution analysis (AEDA), botanical Lujiu, Jiang‐flavor Baijiu, medicine and food of the same origin, sensory evaluation

## Abstract

This study successfully developed a botanical‐type Lujiu product based on Jiang‐flavor Baijiu (BTLJF) with a distinctive flavor profile. The aroma composition was systematically investigated using GC‐O‐MS, AEDA, and traceability analysis. A reliable quantitative method was optimized to ensure high sensitivity. A total of 83 volatile compounds were identified by GC–MS, including 18 esters, 14 alcohols, 1 aldehyde, 6 acids, 3 ketones, 2 acetals, 13 aromatic compounds, 3 terpenes, and 23 heterocyclic compounds. Among these, 48 aroma‐active compounds were further recognized by GC‐O, and 25 compounds with flavor dilution (FD) factor ≥ 9 were quantified as key aroma contributors based on their high odor activity values (OAVs). Seventeen compounds with the highest OAVs, were identified as pivotal constituents shaping the overall aroma profile, including ethyl isovalerate, ethyl butanoate, 2,3‐butanediol, 1‐propanol, phenylacetic acid, butyric acid, ethyl phenylacetate, 3‐methylbutyric acid, 2‐ethyl‐6‐methylpyrazine and acetic acid. Notably, 44 compounds showed a significant positive correlation (*p* < 0.05) with the dosage of medicinal prescriptions. Furthermore, key terpenoids contributing to the herbal aroma in BTLJF, such as DL‐Limonene, (−)‐α‐cedrene, cedrol, nerolidol and α‐terpineol, were confirmed to originate from medicinal prescriptions. This study provides valuable insights for the future improvement of the flavor and quality of Lujiu products.

## Graphical abstract

A novel botanical Lujiu with distinctive flavor characteristics was successfully developed based on Jiang‐flavor baijiu through laboratory‐scale production. Optimized quantitative analysis identified key aroma‐active compounds through a sensomics approach, and a high‐sensitivity method was validated. Traceability analysis linked aroma constituents to medicinal prescriptions, elucidating their role in shaping the aroma wheel and functional properties.

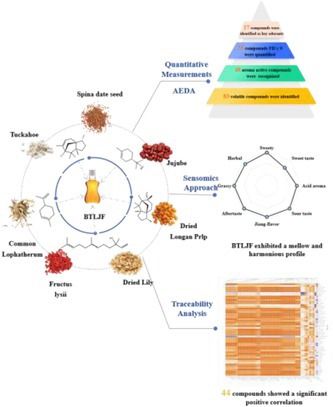

**Abbreviations**
AEDA: aroma extraction dilution analysis
BTLJF: Jiang‐flavor Baijiu
FD: flavor dilution factor
GC‐O‐MS: gas chromatography‐olfactometry‐mass spectrometry
LLME: liquid–liquid microextraction
OAVs: odor activity values
RIs: retention indices

## 1 Introduction

Lujiu is a traditional Chinese liquor with a history spanning over 3500 years, originating in the Shang Dynasty and gaining popularity during the Tang Dynasty (Niu et al. 2022). It integrates elements of traditional Chinese culture, including dietary practices and traditional Chinese medicine. The latest definition of Lujiu is “using huangjiu or baijiu as the base, alcoholic beverages with specific style made by adding substances that are both food and Chinese medicinal materials, or specific raw food materials, or auxiliary materials that comply with relevant regulations, and going through extraction and (or) redistillation, or directly adding certain food extracts, without directly or indirect adding of food additives.” Based on the type of raw materials added, Lujiu is classified into botanical‐type Lujiu, animal type Lujiu, botanical and animal type Lujiu, and other type Lujiu, with botanical‐type Lujiu accounting for approximately 93% of all products (Niu et al. 2022). In response to growing consumer health demands and the diverse health benefits associated with Lujiu, it has gained significant market potential (Zhang et al. 2022). Lujiu has now been recognized as an independent category of liquor in China, alongside fermented alcohol, distilled alcohol, and integrated alcohol, reflecting its increasing importance (China, 2021). The total market value of Lujiu in China is estimated at approximately 50 billion yuan, with projections indicating it could reach 200 billion yuan by 2030. Similar to gin, one of the six primary distilled spirits globally, Lujiu is also made from a variety of healthy food and medicinal ingredients, which contribute to its rich flavor profile and functional value.

Based on the type of raw materials added, botanical‐type Lujiu is particularly appealing to younger consumers due to its fresh aroma and diverse flavor profiles. According to the List of Articles That Are Both Food and Drugs, medicinal and food‐homologous ingredients from classic prescriptions were selected to be incorporated into botanical‐type Lujiu, leveraging their ornamental and health‐promoting properties (Niu et al. 2022). Flavor plays a crucial role in determining the sensory quality of Lujiu and significantly influences consumer preferences. To achieve distinctive flavor characteristics, different base liquors are used in the production of Lujiu, including strong‐flavor, rice‐flavor and Jiang‐flavor, etc. Among these, Jiang‐flavor baijiu is highly favored by consumers for its outstanding sauce flavor, elegant and delicate profile, mellow body, long aftertaste and the persistent aroma in the empty cup (Wang et al. 2024). The BTLJF not only integrates the unique flavor of Jiang‐flavor baijiu with the health benefits and herbal aroma of medicinal prescriptions (Huang 2007), but also exhibits a distinct style and outstanding sensory characteristics, making it an attractive option for consumers who lappreciate both Jiang‐flavor baijiu and Lujiu (Guo et al. 2024).

Over the past decades, research on traditional alcohol genres such as Baijiu, Huangjiu and beer has been relatively deep and comprehensive in areas such as flavor analysis, manufacturing process, fermenting microbiota, storage and aging (Li, Zhang, et al. 2023). However, systemic research on Lujiu, especially regarding its flavor characteristics, remains relatively limited. The main goals of this study were to (1) characterize the key aroma compounds of BTLJF using sensory evaluation, aroma extract dilution analysis (AEDA) and odor activity value (OAV); (2) optimize the liquid–liquid microextraction (LLME‐GC/MS) method for the determination and quantification of the key aroma‐active compounds; (3) explore the origin of important aroma compounds in BTLJF.

## 2 Materials and Methods

### 2.1 Chemicals

All chemical standards were chromatographic grade with at least 97% purity. The following compounds were obtained from Sigma China Co. (Shanghai, China): Ethyl butyrate (≥ 98%), 1‐Propanol (≥ 98%), 3‐methyl‐1‐butanol (≥ 99%), cyclopentanol (≥ 99%), acetic acid (≥ 98%), Propionic acid (≥ 98%), 3‐ethyl‐3‐pentanol (≥ 97%), tetramethylpyrazine (≥ 99.8%), butyric acid (≥ 98%), 3‐methylbutyric acid (≥ 99%), ethyl phenylacetate (≥ 98%), phenethyl acetate (≥ 99%), phenethyl alcohol (≥ 99%), γ‐nonanolactone (≥ 97%), diethyl ester of butanedioic acid (≥ 98%), ethyl 3‐phenylpropionate (≥ 98%), furfural (≥ 97%), benzaldehyde (≥ 98%), ethyl benzoate (≥ 97%), phenylacetic acid (≥ 97%), 5‐methyl furfural (≥ 98%), 5‐methyl‐2‐acetylfuran (≥ 97%), benzyl alcohol (≥ 97%), ethyl levulinate (≥ 98%), 2‐ethyl‐6‐methylpyrazine (≥ 97%), 1‐phenylbutan‐2‐ol (≥ 98%), D‐limonene (≥ 98%), cedrol (≥ 97%), (−)‐α‐cedrene (≥ 97%), eucalyptol (≥ 97%), α‐terpineol (≥ 98%), α‐caryophyllene (≥ 98%), alloaromadendrene (≥ 97%), eremophilene (≥ 98%), nerolidol (≥ 98%), hexanoic acid (≥ 98%). The following compounds were obtained from Tedia China Co. (Shanghai, China): ethyl acetate (HPLC grade) and dichloromethane (HPLC grade). The following compounds were obtained from China National Pharmaceutical Group Corp. (Shanghai, China): *N*‐pentane (HPLC grade), hexane (HPLC grade), anhydrous diethyl ether (AR grade), sodium sulfate (AR grade), sodium chloride (AR grade). Pure water was obtained from a Milli‐Q purification system (Millipore, Bedford, MA). A C5‐C30 n‐alkane mixture (Sigma‐Aldrich) was used for determining the linear retention indices (RIs) of flavor compounds. Other chemicals and reagents were of analytical grade.

Jiang‐flavor base wine was provided by Guizhou Maotai Winery (Group) Health Wine Co. Spina date seed, Jujube, Tuckahoe, Dried Longan Prlp, Common Lophatherum, Fructus lysii, Dried Lily were provided by Tongrentang Pharmaceutical Factory, Beijing Tongrentang.

### 2.2 Samples

The production process for BTLJF was as follows: seven drugs of medicine and food homologous drugs with a certain ratio were selected in classic prescriptions. The dosage of the prescriptions is 2% of the Jiang‐flavor base Baijiu. All samples were soaked in Jiang‐flavor base Baijiu for 40 days in Tongrentang Pharmaceutical Factory, Beijing Tongrentang. After soaking, all the samples were filtered by filtration equipment. Lujiu products were stored at room temperature, protected from light prior to analysis.

Experimental samples for traceability analysis of flavor substances were performed as follows: In 250 mL screw‐top bottles, 100 mL of a 53%vol aqueous alcohol solution and 1, 2, 5, 10, 20 g of the grouped medicinal prescriptions were added and, after 40 days of soaking, filtered through medium‐speed filter paper. All samples above were performed in triplicate, and then stored at room temperature, protected from light prior to analysis.

### 2.3 Sensory Analysis

To select the representative aroma descriptors of BTLJF, the experiment was conducted according to GB/T 16861‐1997 with modifications (Sun et al. 2018). A 10‐member sensory evaluation team, composed of experienced panelists from the Flavor Research Laboratory of the Technical Center in Kweichow Moutai Co. Ltd. participated in the assessment. Each sample (15 mL) was poured into a 3‐digit coded tulip‐type glass and presented to the panelists. The panelists were required to write down at least 10 sensory descriptors for the BTLJF sample and rate the intensity of each descriptor on a 0–5 scale (0 = none, 1 = very weak, 2 = weak, 3 = medium, 4 = strong, 5 = very strong). The evaluation was performed in three replicates. The M value for each sensory descriptor was calculated based on the following formula (GB/T 16861‐1997).

$$M=\sqrt{I\times F}$$

where *F* represents the percentage of the number of times the descriptor is actually mentioned in its total number of possible mentions; *I* represents the percentage of the actual strength given by the panel in the maximum possible strength of the descriptor. In this study, the top 8 descriptors with the highest *M* values were selected for sensory evaluation as these descriptors exhibit the greatest contribution to the overall sensory profile of the product.

### 2.4 Isolation of the Volatile Compounds

Volatile compounds in the Lujiu sample were extracted using the Liquid–liquid microextraction (LLME) method with modifications (Du et al. 2021). The sample (52% BTLJF) was diluted to 10% ethanol (v/v) with ultra‐pure water, and sodium chloride was added to the solution to saturation. The solution was then extracted three times with ethyl acetate (50 mL each time, twice) and diethyl ether (50 mL each time, once). The organic phase was collected and dried over anhydrous sodium sulfate overnight. The extract was concentrated to 1.0 mL using a vacuum concentrator at 40°C and stored at −20°C prior to GC‐O and GC–MS analysis.

Aroma compounds were identified by comparing their mass spectra (MS) and retention indices (RIs) with those in the NIST 2017 database and the internal database of our laboratory. The MS results and odors of the detected compounds were further compared with corresponding standards (Sun et al. 2017).

### 2.5 GC‐O and Aroma Extract Dilution Analysis (AEDA)

GC‐O‐MS analysis was performed on an Agilent 7890A gas chromatograph equipped with an Agilent 5975C mass‐selective detector and a sniffing port (ODP 2, Gerstel, Germany). The concentrated solution were separated using a DB‐WAX column (30 m × 0.25 mm i.d., 0.25 μm film thickness; Agilent, Torrance, CA). This procedure was a modification of a reported method (Hofmann et al. 2008). The inlet temperature was 250°C in splitless mode. Helium (purity 99.999%) was used as the carrier gas at a constant flow rate of 1.0 mL/min. The oven temperature was initially 40°C for 1 min, then ramped to 130°C at 3°C/min, and further to 220°C at 10°C/min, with a final hold time of 10 min. The mass spectrometer was operated in electron ionization (EI) mode, with electron energy of 70 eV, ion source temperature of 230°C, and quadrupole temperature of 150°C. The sniffing port temperature was set to 230°C. The retention index (RI) was calculated based on the retention time of each aroma compound. Odor identification was performed by comparing the odor descriptors, RI, and mass spectra of the detected compounds with those of pure reference standards.

The concentrated extract was diluted 3‐fold stepwise with diethyl ether and a 1 μL volume was injected into the GC injector in splitless mode. After each injection, the aroma was sniffed and the characteristics and retention time of each aroma compound were recorded by the perfumer until no odor was detectable. Based on the average result of the panelists, each aroma‐active compound was assigned a flavor dilution (FD) factor. The maximum dilution of sniffed aroma compounds was taken to be the FD value, indicating the contribution of the compound to the overall aroma profile of BTLJF. The experiment was repeated three times by each panelist.

### 2.6 Qualitativez and Quantitative Analysis of the Flavor Compounds

Three methods were used to quantify the concentrations of the aroma compounds.

1. For compounds with high concentrations, direct injection with gas chromatography‐flame ionization detection (GC‐FID) was employed, (Agilent 7890; Agilent Technologies, USA). This method was based on a previously reported procedure (Li, Wang, et al. 2023) with several modifications. Each sample (1.0 μL) was separated on a DB‐WAX UI capillary column (30 m × 0.25 mm, 0.25 μm, Agilent Technologies, USA). The oven temperature was initially set at 40°C and held for 4 min, then ramped to 100°C at 5°C/min, and further increased to 200°C at 10°C/min, with a final hold time of 18 min. Helium (purity 99.999%) was used as the carrier gas at a constant flow rate of 1.0 mL/min. The inlet temperature was set at 250°C, the detector temperature was set at 300°C, and the split ratio was set at 30:1. About five compounds were quantified using this method.
2. For trace compounds, liquid–liquid microextraction (LLME) combined with GC–MS was used. This method was based on a previously reported procedure (Liu et al. 2023; Ortega et al. 2001), with some modifications. The experimental samples was diluted to an appropriate ethanol (v/v) with saturated sodium chloride. Then, 10 mL diluted sample was transferred to a 20 mL screw‐capped headspace bottle and 2 μL of citronellol (11,727 mg/L) was added as an internal standard. The organic solvent was extracted with 1 mL diethyl ether ethyl. After static layering, the upper organic phase was transferred to an injection vial (2 mL) for GC/MS analysis. The injection volume was 1.0 μL, and the inlet temperature was 230°C. Other instrument conditions were consistent with those described in 2.5. About 20 compounds were quantified using this method.
3. Headspace solid phase microextraction (HS‐SPME) combined with GC–MS was also employed to characterize and quantify the terpenes in BTLJF, based on the method used in a previous studies (Wang et al. 2016; Fan and Qian 2005) The BTLJF samples were diluted with ultrapure water to a final concentration of 10% (v/v) ethanol. A 10 mL aliquot of the diluted sample was transferred to a 20 mL screw‐capped headspace bottle and saturated with 3 g NaCl. Two microliters of menthol (56.25 μg/L) was added as an internal standard. An automatic headspace sampling system (MultiPurpose Sample MPS 2 with a SPME adaptor, from Gerstel Inc., Mülheim, Ruhr, Germany) with a 50/30 μm divinylbenzene/carboxen/polydimethylsiloxane (DVB/CAR/PDMS) fiber (2 cm, Supelco Inc., Bellefonte, PA, USA) was used to extract the volatile compounds. The extraction conditions were as follows: the sample was equilibrated at 60°C for 5 min, followed by 40 min of extraction under stirring at 250 rpm. After extraction, the fiber was desorbed into the GC injection port at 250°C for 5 min.

### 2.7 OAV Analysis

The OAVs of the aroma compounds in BTLJF were calculated based on their concentrations in the sample and their corresponding thresholds in the alcohol solution. In general, compounds with OAVs ≥ 1 are considered to contribute significantly to the overall aroma profile of BTLJF (Wang et al. 2020). The odor detection thresholds in this study were derived from previous research (Van Helmert 2018). The matrix for the measurement of these thresholds was described in Table 3.

### 2.8 Traceability Analysis of Important Aroma‐Active Compounds

Experimental samples for traceability analysis of flavor substances were analyzed with LLME‐GC‐MS and HS‐SPME methods, as described in Section 2.6. The correlation between aroma flavor compounds and dosage of medicinal prescriptions was evaluated using SPSS. To identify the source of the flavor in BTLJF, target compounds were identified as those whose flavor intensity increased with the amount of prescription added, and comparative analysis was performed to determine whether these compounds were present in the Jiang‐flavor base baijiu used in the production.

### 2.9 Statistical Analysis

The data and correlation analysis were performed by using Origin (2025) and IBM SPSS Statistics 23.

## 3 Results and Discussion

### 3.1 The Aroma Profile of the BTLJF Product

**FIGURE 1:**
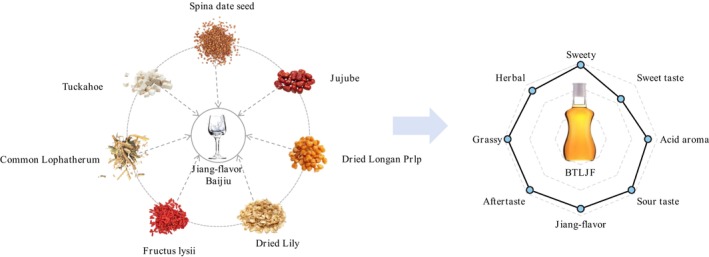
Botanical‐type Lujiu product based on Jiang‐flavor Baijiu (BTLJF).

The BTLJF product was transparent and golden in color, with no visible suspensions (Figure 1). It exhibited a mellow and harmonious profile, characterized by an exceptional herbal flavor and an elegant, well‐coordinated fragrance. Eight descriptors (sweety, grassy, aftertaste, herbal, acid aroma, sour taste, jiang‐flavor, sweet taste) were selected to describe the aroma characteristics of the BTLJF product, based on their usage frequency and perceived intensity. The aroma intensity for each descriptor was determined by averaging the scores provided by the sensory panel, and the results are shown in Table 1. The overall aroma characteristics of BTLJF product was primarily described as an obvious herbal aroma and grassy aroma, outstanding Jiang‐flavor and sweety aroma, medium acid aroma with long aftertaste and pleasant sweet and sour taste.

**TABLE 1:** Aroma descriptors of BTLJF and their *M* values.

No.	Aroma descriptors	Frequency ratio	Strength ratio	*M* value
1	Sweety	1	0.58	0.76
2	Grassy	1	0.57	0.76
3	Aftertaste	1	0.56	0.75
4	Herbal	1	0.54	0.73
5	Acid aroma	1	0.53	0.73
6	Sour taste	0.91	0.56	0.72
7	Jiang‐flavor	0.91	0.55	070
8	Sweet taste	1	0.45	0.67

### 3.2 Aroma‐Active Compounds Determined in BTLJF by GC‐O‐MS and AEDA Analysis

The BTLJF was analyzed using LLME‐GC/MS, and a total of 83 volatile compounds were identified, including 18 esters, 14 alcohols, 1 aldehyde, 6 acids, 3 ketones, 2 acetals, 13 aromatic compounds, 3 terpenes, and 23 heterocyclic compounds. The extract from Section 2.5 was further analyzed using GC‐O‐MS and AEDA to identify the aroma‐active components. All aroma‐active compounds were determined by comparing their MS and RIs with those in the NIST (2017), and by comparing their aroma with reference standards. As shown in Table 2, a total of 48 aroma‐active regions with FD factors ranging from 3 to 81 were detected in BTLJF samples, Of these, 47 were further identified based on their MS, RIs, and aroma characteristics, including 11 esters, 6 alcohols, 1 aldehyde, 6 acids, 14 aromatics, and 9 heterocyclic compounds (Table 2). Since BTLJF is based on Jiang‐flavor baijiu, the aroma‐active compounds are expected to resemble those of Jiang‐flavor baijiu.

**TABLE 2:** Aroma‐active compounds in BTLJF identified by LLME coupled with GC‐O‐MS.

No.	RI^a^		Descriptor	Aroma compounds	FD factors	Basis of identification^b^
	DB‐WAX‐UI	DB‐5				
1	1005	782	Fruity	Isobutyl acetate	3	MS, RI, odor
2	950	728	Fruity	Propyl acetate	27	MS, RI, odor
3	1064	854	Fruity	Ethyl Isovalerate	9	MS, RI, odor
4	1049	802	Fruity	Butyl acetate	27	MS, RI, odor
5	1035	800	Fruity	Ethyl butyrate	81	MS, RI, odor
6	1044	790	Fruity	Isopentyl formate	3	MS, RI, odor
7	1033	560	Grassy	1‐Propanol	81	MS, RI, odor
8	1071	803	Grassy	Hexanal	3	MS, RI, odor
9	1134	668	Grassy	1‐Butanol	3	MS, RI, odor
10	1085	622	Grassy	2‐Methyl‐1‐propanol	3	MS, RI, odor
11	1249	993	Minty	2‐Pentylfuran	3	MS, RI, odor
12	1217	747	Grassy	3‐Methyl‐1‐butanol	81	MS, RI, odor
13	1274	769	Grassy	1‐Pentanol	3	MS, RI, odor
14	1443	600	Vinegar	Acetic acid	81	MS, RI, odor
15	1457	954	Caramel	2‐Methylpropyl 2‐hydroxypropanoate	3	MS, RI, odor
16	1382	991	Roasted aroma	2‐ethyl‐6‐methylpyrazine	9	MS, RI, odor
17	1478	832	Baking aroma	Furfural	27	MS, RI, odor
18	1485	1086	Roasted aroma	Tetramethylpyrazine	81	MS, RI, odor
19	1530	701	Vinegar	Propionic acid	27	MS, RI, odor
20	1547	1064	Sweety	Ethyl DL‐Leucate	3	MS, RI, odor
21	1489	910	Caramel	2‐Acetylfuran	3	MS, RI, odor
22	1550	791	Vinegar	Isobutyric acid	3	MS, RI, odor
23	1527	962	Nutty	Benzaldehyde	27	MS, RI, odor
24	1563	790	Cucumber Aroma	2,3‐Butanediol	9	MS, RI, odor
25	1570	957	Sesame aroma	5‐Methyl furfural	9	MS, RI, odor
26	1607	1020	Sweety	Ethyl levulinate	9	MS, RI, odor
27	1611	823	Cheesy, creamy	Butyric Acid	81	MS, RI, odor
28	1597	1037	Baking aroma	5‐Methyl‐2‐acetylfuran	9	MS, RI, odor
29	1630	940	Sweety	Gamma Butyrolactone	3	MS, RI, odor
30	1644	1171	Rose	Ethyl benzoate	9	MS, RI, odor
31	1680	831	Stinky sour	3‐Methybutyric Acid	81	MS, RI, odor
32	1672	1179	Fruity	Diethyl ester of butanedioic acid	9	MS, RI, odor
33	1683	1186	Sweety	lactone	9	MS, RI, odor
34	1785	1243	Rose	Ethyl phenylacetate	81	MS, RI, odor
35	1791	1255	Sweety	Phenethyl acetate	81	MS, RI, odor
36	1854	999	Cheesy, fatty	Hexanoic acid	9	MS, RI, odor
37	1897	1033	Floral	Benzyl alcohol	9	MS, RI, odor
38	1900	1375	Sweety	Ethyl 3‐phenylpropionate	9	MS, RI, odor
39	1939	1113	Rose	Phenethyl alcohol	81	MS, RI, odor
40	1900	1015	Sweety	1‐Phenylbutan‐2‐ol	9	MS, RI, odor
41	1984	970	Herbal	Phenol	3	MS, RI, odor
42	2018	1344	Sweety	γ‐Nonanolactone	27	MS, RI, odor
43	2014	1270	Sweety	Diethyl malate	3	MS, RI, odor
44	2078	1069	Pill aroma	p‐Cresol	9	MS, RI, odor
45	2575	1265	Rose	Phenylacetic acid	9	MS, RI, odor
46	2633	1343	Rose	3‐Phenylpropionic acid	3	MS, RI, odor
47	2585	1391	Vanilla	Vanillin	3	MS, RI, odor
48	1700	1226	Baking aroma	2,3‐Dihydrobenzofuran	3	MS, RI, odor

Abbreviation: FD, flavor dilution factor.

^a^ RI: retention index of different stationary phases.

^b^ Identification based on MS (mass spectrometry), RI (retention index) and odor description (Cates and Meloan 1963).

Aromatic compounds with floral and nutty notes were among the most abundant flavors in BTLJF. Approximately 10 aromatic compounds exhibited FD values of ≥ 9, indicating their significant contribution to the overall aroma profile of BTLJF. Among these, ethyl phenylacetate, phenethyl acetate and phenethyl alcohol showed the highest FD values (FD = 81), corresponding to rosy and floral aromas, suggesting their potential importance in the aroma of BTLJF. Other notable compounds included benzaldehyde (FD = 27, nutty), ethyl benzoate, ethyl 3‐phenylpropionate and 1‐phenylbutan‐2‐ol (FD = 9, sweet and honey), benzyl alcohol (FD = 9; rosy and floral), and p‐cresol (FD = 9, herbal and pill aroma).

Esters with fruity notes were one of the most abundant flavors in BTLJF, with ethyl esters constituting the majority. Among them, ethyl butanoate, which imparts a fruity aroma, exhibited the highest FD value of 81, followed by isobutyl acetate and butyl acetate (FD = 27, fruity), and ethyl isovalerate, ethyl DL‐leucate, diethyl ester of butanedioic acid and ethyl levulinate (FD = 9, fruity and sweet). These esters are primarily formed through esterase‐catalyzed condensation reactions between alcohols and carboxylic acids (Xu 2019).

Heterocyclic compounds in BTLJF primarily contributed to baking and sweet aroma. Notably, tetramethylpyrazine exhibited the highest FD factor (81; roasted and nutty), corresponding to roasted and nutty notes, indicating its significant role in the aroma of BTLJF. Nitrogen‐containing heterocycles with such sensory characteristics are typically formed through protein degradation via acid hydrolysis (Niu et al. 2016). Furfural (FD = 27, baking) and γ‐nonanolactone (FD = 27, sweet and honey) were also identified as important contributors. Additionally, 2‐ethyl‐6‐methylpyrazine, 5‐methyl‐2‐acetylfuran (FD = 9, roasted and baking) and 5‐methyl furfural (FD = 9, sesame aroma) were detected and confirmed.

Acids played a crucial role in shaping the flavor profile of BTLJF, as their absence in Baijiu can lead to a harsh and unbalanced mouthfeel (Xu 2019). Key acids detected in BTLJF included acetic acid, butyric acid, 3‐methylbutyric acid (FD = 81; vinegar and stinky sour), propionic acid (FD = 27, acid), and hexanoic acid (FD = 9, cheesy, fatty). These compounds exhibited high FD factors, indicating their significant contribution to the overall aroma and taste. Previous studies suggest that these acids are primarily derived from amino acids such as alanine, 3‐methylbutanoic acid from leucine or isoleucine, and phenylacetic acid from phenylalanine (Glomb 2017).

Alcohol compounds in BTLJF primarily contributed to the grassy and plant‐like aromas, which were predominantly generated through the decarboxylation and dehydrogenation of amino acids during fermentation or sugar metabolism (Hazelwood et al. 2008). These compounds were identified as important contributors to the overall flavor and harmony of the BTLJF. Notably, 1‐propanol and 3‐methyl‐1‐butanol showed the highest FD values (FD = 81; grassy and plant‐like), indicating their potential importance in defining the aromatic characteristics of BTLJF. Additionally, 2,3‐Butanediol (FD = 9, cucumber‐like) demonstrated the second FD factor, further emphasizing its contribution to the flavor complexity.

Hexanal (FD = 3), characterized by a distinct grassy aroma, was the only aldehyde compound identified in BTLJF. Traceability analysis revealed that its presence was primarily derived from the medicinal prescription components.

### 3.3 Optimization of LLME‐GC/MS Analysis Parameters

**FIGURE 2:**
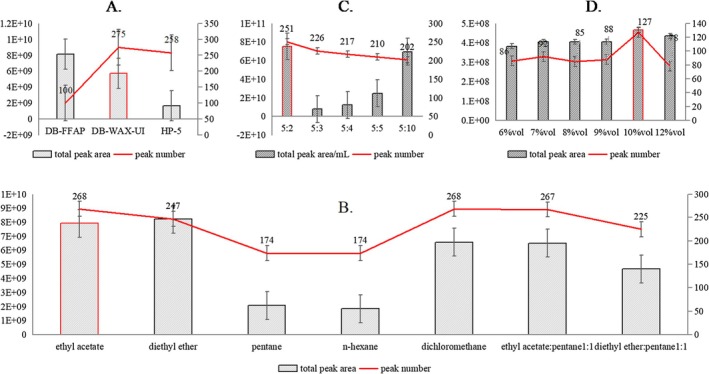
Optimization of LLME‐GC/MS analysis parameters: chromatographic columns (A); extraction solvent (B); material‐liquid ratio (C); ethanol concentration (D).

Since there has been no established quantitative analysis method for BTLJF, several key parameters were optimized through single‐factor experiments to improve the extraction efficiency of flavor compounds. These parameters included the type of chromatographic column, the extraction solvent, the ethanol content, and the material‐liquid ratio. As shown in Figure 2, the number and peak areas of detected compounds were used as evaluation indicators to assess the performance of different experimental conditions.

To comprehensively analyze the flavor compounds in BTLJF, it is essential to detect a large number of flavor substances with high sensitivity. Therefore, various polar chromatographic columns were evaluated. Although the DB‐FFAP column exhibited the highest total peak responses, only 100 flavor substances were identified. In contrast, the DB‐WAX‐UI column enabled the detection of more flavor compounds, making it more suitable for evaluating the overall flavor profile of BTLJF. Based on the combined analysis of peak number and area, the DB‐WAX‐UI column was selected as the optimal choice for the GC–MS analysis of BTLJF.

For solvent optimization, different solvents and solvent mixtures were tested for extraction efficiency. The results indicated that medium‐polarity solvents such as ethyl acetate and the mixed solvent system (ethyl acetate:pentane = 1:1, v/v) demonstrated superior performance in terms of both the number of extracted compounds and the total peak area compared to other solvents.

The material‐to‐liquid ratio was also systematically optimized. The optimal ratio was found to be 5:2 (w/v), which yielded the highest number of flavor compounds and total peak area. Additionally, the effect of ethanol concentration on extraction efficiency was investigated by testing five different levels (6%, 7%, 8%, 9%, 10%, and 12%). The results showed that the number of detected compounds and the total peak area initially increased with increasing ethanol concentration, reaching a maximum at 10% ethanol, and then decreasing at higher concentrations. Therefore, 10% ethanol was determined to be the optimal dilution level for the extraction of flavor compounds in BTLJF.

### 3.4 Quantitation and OAV Calculation of Major Aroma‐Active Compounds in BTLJF

To assess the contributions of aroma‐active compounds to the overall aroma profile of BTLJF, approximately 25 compounds with FD ≥ 9 were quantified using a standard calibration curve (Table 3). The results indicated that 2,3‐butanediol (1935.02 mg/L) was the most abundant, followed by acetic acid (1907.36 mg/L), 1‐propanol (1201.00 mg/L), 3‐methyl‐1‐butanol (241.94 mg/L) and furfural (110.11 mg/L). All these compounds exceeded a concentration threshold of 100 mg/L, suggesting their potential significance as key volatile constituents of BTLJF. In addition, butanoic acid, ethyl ester, phenylacetic acid, butyric acid, and 3‐methylbutyric acid were also detected at relatively high concentrations (≥ 10 mg/L). Smaller quantities of other compounds were also identified.

**TABLE 3:** Quantification and OAVs of major aroma‐active compounds (FD ≥ 9) in BTLJF by GC–MS and GC.

No.	Compounds	*R* ^2^	Recovery ratio	RSD (%)	Concentration^c^ (mg/L)	Odor threshold (μg/L)	OAV	Method	Extracting solvent
								GC/GC–MS	GC
1	Butanoic acid, ethyl ester	0.999	97.41	0.07	36.86	81.5^a^	452.304	GC	/
2	Acetic acid	0.999	94.73	0.07	1907.36	160,000.0^a^	11.921	GC	/
3	Propionic acid	0.998	84.73	0.85	24.97	8100.0^a^	3.082	GC	/
4	Butyric Acid	0.999	83.04	1.79	17.35	964.6^a^	17.981	GC	/
5	Furfural	0.998	119.90	4.159	110.11	44,029.7^a^	14.913	GC	/
6	1‐Propanol	0.999	85.22	3.7	1201.00	53,952.6^a^	2.316	GC	/
7	Butanoic acid, 3‐methyl—	0.999	89.17	2.19	15.59	1045.5^a^	0.618	GC–MS	Ethyl acetate
8	γ‐Nonanolactone	0.999	86.36	7.692	0.21	90.7^a^	15.117	GC–MS	Ethyl acetate
9	Phenethyl alcohol	0.994	115.10	6.996	17.86	28,922.7^a^	0.006	GC–MS	Ethyl acetate
10	Ethyl phenylacetate	0.997	87.31	0.236	6.15	406.8^a^	2.396	GC–MS	Ethyl acetate
11	Diethyl ester of butanedioic acid	1.000	81.03	6.062	1.95	353,193.3^a^	2.501	GC–MS	Ethyl acetate
12	Ethyl 3‐phenylpropionate	0.999	89.20	15.625	0.30	125.2^a^	0.807	GC–MS	Ethyl acetate
13	Benzaldehyde	0.996	86.47	0.01	3.39	4203.1^a^	0.105	GC–MS	Ethyl acetate
14	Ethyl benzoate	0.991	128.61	0.035	0.15	1433.7^a^	1.278	GC–MS	Ethyl acetate
15	Phenethyl acetate	0.996	120.30	0.016	0.52	406.8^a^	19.958	GC–MS	Ethyl acetate
16	Phenylacetic acid	0.990	96.04	0.124	28.54	1430.0^a^	0.001	GC–MS	Ethyl acetate
17	5‐Methyl furfural	0.999	95.22	0.052	0.57	466,321.1^a^	0.015	GC–MS	Ethyl acetate
18	5‐Methyl‐2‐acetylfuran	0.992	95.52	0.051	0.61	40,870.1^a^	0.014	GC–MS	Ethyl acetate
19	Benzyl alcohol	0.996	105.03	0.056	0.56	40,927.2^a^	30.579	GC–MS	Ethyl acetate
20	2,3‐Butanediol	0.997	99.80	0.213	1935.02	63,280.0^a^	1.350	GC–MS	Ethyl acetate
21	3‐Methyl‐1‐butanol	0.991	84.33	4.000	241.94	179,190.8^b^	0.002	GC–MS	Ethyl acetate: Pentane = 1:1
22	Ethyl levulinate	0.998	83.92	3.85	0.52	225,164.4^a^	13.313	GC–MS	Ethyl acetate: Pentane = 1:1
23	2‐Ethyl‐6‐methylpyrazine	0.998	83.93	1.8	0.53	40.0^a^	1.325	GC–MS	Ethyl acetate:Pentane = 1:
24	Hexanoic acid	0.992	93.90	18.7	3.34	2517.2^a^	22.260	GC–MS	Ethyl acetate: Pentane = 1:1
25	Ethyl isovalerate	0.999	94.02	7.51	5.58	6.9^a^	809.869	GC–MS	Ethyl acetate: Pentane = 1:1

^a^ Reported threshold values for this compound are available in the literature (Ma 2019).

^b^ The threshold of this compound was determined for the first time in this study.

^c^ The concentrations of aroma‐active compounds were precisely determined by quantitative analysis.

To further evaluate the contributions of odorants to the overall aroma profile of BTLJF, the odor activity values (OAVs) of the aroma‐active compounds were calculated (Table 3). Seventeen compounds with OAVs > 1 were identified as key aroma‐active compounds in BTLJF. Among these, ethyl isovalerate (809), butanoic acid, ethyl ester (452) exhibited the highest OAVs, exceeding 100, indicating their significant contribution to the overall aroma. Additionally, 2,3‐butanediol (31), 1‐propanol (22), phenylacetic acid (20), butyric acid (18), ethyl phenylacetate (15), butanoic acid, 3‐methyl‐ (15), 2‐ethyl‐6‐methylpyrazine (13) and acetic acid (12) also showed OAVs greater than 10, further highlighting their importance in shaping the aroma of BTLJF. Other compounds with moderate OAVs, such as propionic acid (3), γ‐nonanolactone, ethyl 3‐phenylpropionate and furfural (2), as well as phenethyl acetate, 3‐methyl‐1‐butanol, hexanoic acid (1), were also identified and may contribute to the unique characteristics of BTLJF.

### 3.5 Traceability Analysis of Important Aroma‐Active Compounds

**FIGURE 3:**
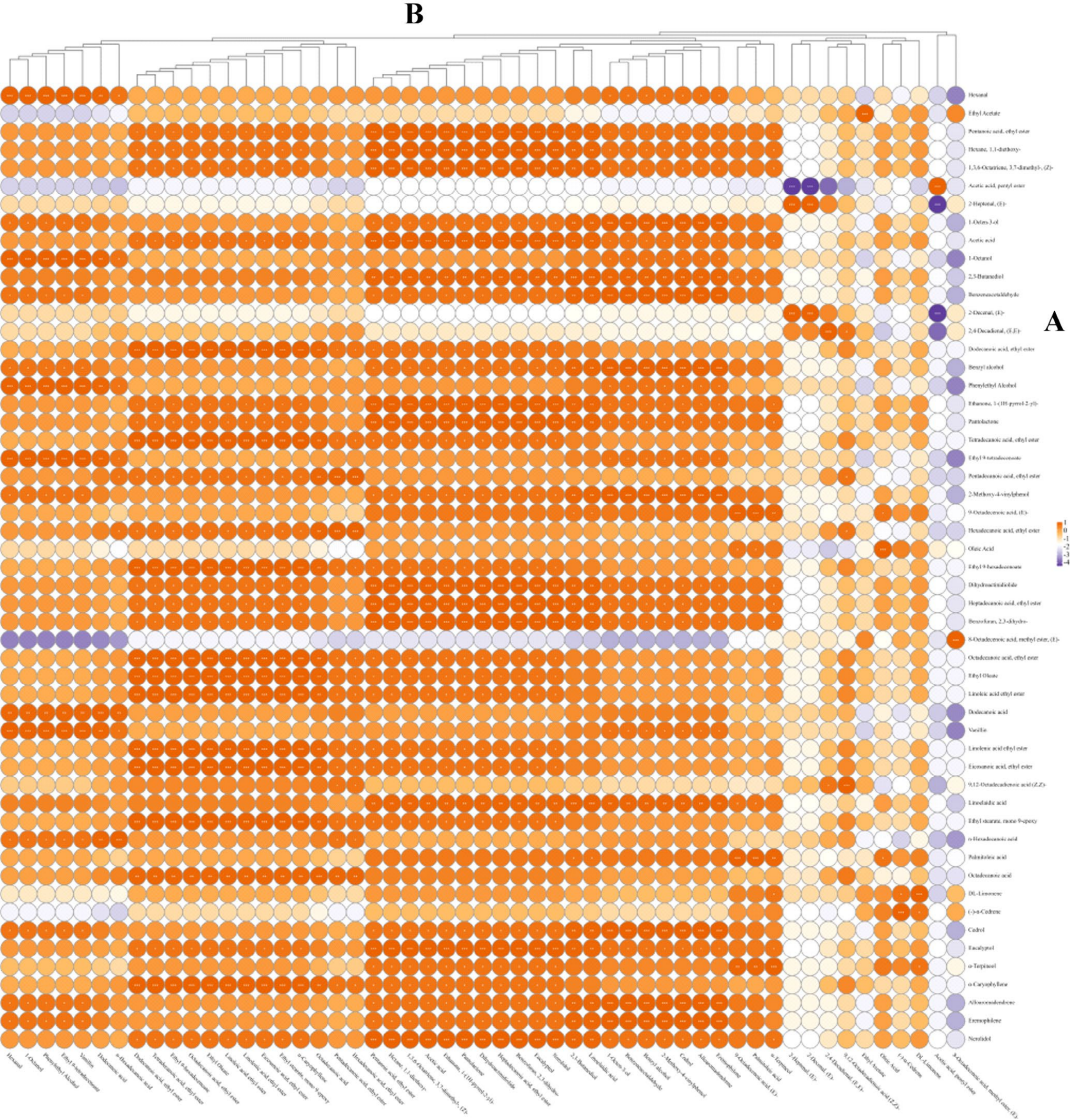
(A) Qualitative compounds in BTLJF products and experimental samples; (B) Correlation analysis between dosages of medicinal prescriptions and flavor compounds.

To better control the flavor quality of BTLJF, it is essential to identify the source of its aroma‐active compounds. Comparative analysis of aroma compounds in experimental samples prepared from 53%vol ethanol aqueous solutions was performed. A total of 55 compounds were identified in the experimental samples, comprising 18 esters, 3 alcohols, 4 aldehydes, 10 acids, 1 acetal, 9 terpenes, 4 heterocycles, 5 aromatics, and 1 other compound. Of these, 26 substances were detected in the BTLJF product, indicating their contribution to the overall aroma profile. Correlation analysis revealed that 44 compounds showed a significant positive correlation with the dosage of medicinal prescriptions at the *p* < 0.05 level, as shown in Figure 3. The aroma profiles of 19 of these compounds were further characterized, and it was found that they were mainly aldehydes for grassy aroma, terpenes for herbal aroma, and aromatics for floral aroma, as shown in Table 4. Notably, the five terpenes were not detected in the base Jiang‐flavor baijiu, suggesting that the terpenes with herbal flavor in BTLJF originated from the medicinal prescriptions used in the production of BTLJF. This finding highlights that the medicinal prescriptions not only underpin the therapeutic properties of the Lujiu but also serve as a major origin of flavor compounds. Therefore, a scientific investigation into the optimal dosage and ratio of grouped medicinal prescriptions is necessary to ensure the production of BTLJF with distinctive and consistent flavor characteristics.

**TABLE 4:** Aroma compounds in medicinal prescriptions identified by GC‐O‐MS.

No.	RI^a^		Compounds	Aroma descriptor	Basis of identification^d^
	DB‐WAX‐UI	DB‐5			
1	1088	799	Hexanal	Grassy	MS, RI, odor
2	1128	897	Pentanoic acid, ethyl ester	Fruity	MS, RI, odor
3	1310	954	2‐Heptenal, (E)—	Grassy	MS, RI, odor
4	1576	1076	1‐Octanol	Mushroom	MS, RI, odor
5	1448	975	1‐Octen‐3‐ol	Mushroom	MS, RI, odor
6	1520	955	Benzaldehyde	Nutty	MS, RI, odor
7	1810	1310	2,4‐Decadienal, (E,E)—	Oily	MS, RI, odor
8	1966	1060	Ethanone, 1‐(1H‐pyrrol‐2‐yl)—	Onion	MS, RI, odor
9	1203	1025	DL‐Limonene	Woody	MS, RI, odor
10	1579	1412	(−)‐α‐Cedrene	Woody	MS, RI, odor
11	2110	1604^a^	Cedrol	Herbal	MS, RI, odor
12	1415	600	Acetic acid	Vinegar	MS, RI, odor
13	1645	1035	Benzeneacetaldehyde	Floral	MS, RI, odor
14	1860	1030	Benzyl alcohol	Floral	MS, RI, odor
15	1905	1112	Phenylethyl Alcohol	Floral	MS, RI, odor
16	2563	1390	Vanillin	Vanilla	MS, RI, odor
17	2200	1295	2‐Methoxy‐4‐vinylphenol	Herbal	MS, RI, odor
18	2005	1528	Nerolidol	Sweety	MS, RI, odor
19	1680	1195^d^	α‐Terpineol	Herbal	MS, RI, odor

^a^ linear retention indices.

^d^ MS, compounds were identified by MS spectra; aroma, compounds were identified by comparison to the reference standards using GC‐O‐MS; RI, compounds were identified on DB‐WAX‐UI or DB‐5 columns by their calculated RIs based on n‐alkanes (C7‐C30) compared with literature; odor, compounds were identified by sensory evaluation team.

## 4 Conclusion

In this study, a novel botanical Lujiu with distinctive flavor characteristics was successfully developed based on Jiang‐flavor baijiu through laboratory‐scale production. The aroma‐active compounds were quantitatively analyzed with optimized parameters, and the key aroma compounds were identified using a sensomics approach. A robust quantitative method for flavor compound analysis in Lujiu was established by optimizing the column type, extraction solvent, ethanol content, and material‐liquid ratio through single‐factor experiments. Approximately 25 compounds with OAVs ≥ 9 were quantified. A total of 83 volatile compounds were identified by GC–MS, and 48 aroma‐active compounds were further recognized by GC‐O. Among these, ethyl isovalerate, butanoic acid, ethyl ester and 15 other compounds with OAVs > 1 were identified as the key odorants of BTLJF. Traceability analysis revealed that 44 compounds exhibited a significant positive correlation (*p* < 0.05) with the dosage of medicinal prescriptions, indicating that the medicinal prescriptions not only underpin the therapeutic properties of the Lujiu but also serve as a major origin of flavor compounds. Therefore, further scientific research is warranted to determine the optimal dosage of medicinal prescriptions in Lujiu production. Overall, this study elucidates the aroma profile and composition of botanical type Lujiu, establishes analytical methods for immersion‐type Lujiu, and provides valuable insights for enhancing the flavor and quality of Lu‐jiu products in the future.

## Author Contributions

Ping Xiang and Fan Yang: conceptualization; Yubo Yang: methodology; Yongsu Li: software; Yongsu Li and Bo han Zhang: validation; Yongsu Li: formal analysis; Yongsu Li: investigation; Fan Yang: resources; Bo Han Zhang: data curation; Yongsu Li: writing – original draft preparation; Yongsu Li: writing – review and editing; Yubo Yang: visualization; Fan Yang: supervision; Yubo Yang: project administration; Ping Xiang: funding acquisition. All authors have read and agreed to the published version of the manuscript.

## Disclosure

Patents: Zhang et al. (2024). China National Intellectual Property Administration.

## Conflicts of Interest

The authors declare no conflicts of interest.

## Data Availability

The data that support the findings of this study are available from the corresponding author upon reasonable request.
